# Developing a Tool for Cognitive Screening in an Older Adult Psychiatric Rehabilitation Ward

**DOI:** 10.1192/bjo.2024.403

**Published:** 2024-08-01

**Authors:** Elle Newton, Yathooshan Ramesh, Omar Carney, Aroop Bhattacharya, Ioana Popescu

**Affiliations:** Barnet, Enfield & Haringey Mental Health Trust, London, United Kingdom

## Abstract

**Aims:**

Cognitive disorders, such as dementia, are a possible comorbidity and an important differential diagnosis to consider in older adults admitted to psychiatric wards with a functional disorder. Whilst cognitive assessment tools (e.g. ACE-III) and neuroimaging (e.g. MRI scans) are well established, there is significant variability in how and when they are used, which can result in inconsistences in their use. The aim was to identify the types of inconsistencies that may occur, and to provide a standardised framework in order for these tools to be used consistently on our functional rehabilitation ward.

**Methods:**

This QIP retrospectively assessed data for all patients discharged over a 7-month period between October 2022 and May 2023, from an older adult functional rehabilitation ward. Clinical notes were reviewed to determine whether a cognitive assessment and neuroimaging had been considered, and if so, whether the assessment or investigation was appropriate and completed without delay. Correspondence to the GP or CMHT was reviewed to determine whether this had appropriate information about the relevant cognitive screening completed, and had included an appropriate follow-up plan. Data collected was checked for accuracy through screening by a second clinician, after which a consensus meeting was held to account for discrepancies.

**Results:**

25 patients were discharged during the 7-month period. 52% were identified as having an issue or delay in their cognitive screening and correspondence; 32% had a delay in completing a cognitive assessment; 32% did not have an appropriate follow-up plan communicated in their discharge summary regarding future monitoring of their cognition; and 8% had a delay in considering or requesting neuroimaging.

**Conclusion:**

Team discussion identified that staff uncertainty relating to the use of cognitive tools and neuroimaging was a significant contributing factor to the issues identified in our results. We subsequently delivered training using a flowchart for doctors, nurses and allied healthcare professionals on the ward, which included information about the benefits and disadvantages of different screening tools and imaging modalities, in order to assist selection of the most appropriate tools on a case-by-case basis. The flowchart included the need for MDT discussion and senior psychiatrist involvement, but aimed to improve team confidence in understanding the rationale for these decisions. Based on the results of our post-intervention data, we will consider adapting the training and flowchart delivered to meet the needs of other older adult services in the trust.

